# Dissemination of Extended-Spectrum-β-Lactamase-Producing *Enterobacter cloacae* Complex from a Hospital to the Nearby Environment in Guadeloupe (French West Indies): ST114 Lineage Coding for a Successful IncHI2/ST1 Plasmid

**DOI:** 10.1128/AAC.02146-20

**Published:** 2021-02-17

**Authors:** Matthieu Pot, Stéphanie Guyomard-Rabenirina, David Couvin, Célia Ducat, Vincent Enouf, Séverine Ferdinand, Gaëlle Gruel, Edith Malpote, Antoine Talarmin, Sébastien Breurec, Yann Reynaud

**Affiliations:** a Transmission, Reservoir and Diversity of Pathogens Unit, Pasteur Institute of Guadeloupe, Les Abymes, France; b Pasteur International Bioresources network (PIBnet), Plateforme de Microbiologie Mutualisée (P2M), Pasteur Institute of Paris, Paris, France; c Laboratory of Clinical Microbiology, University Hospital of Guadeloupe, Pointe-à-Pitre/Les Abymes, France; d Faculty of Medicine Hyacinthe Bastaraud, University of the Antilles, Pointe-à-Pitre, France; e INSERM, Center for Clinical Investigation 1424, Pointe-à-Pitre/Les Abymes, France

**Keywords:** *Enterobacter cloacae* complex, *bla*_CTX-M-15_, IncHI2, one health, ST114, wastewater treatment plant, wildlife

## Abstract

Wastewater treatment plants are considered hot spots for antibiotic resistance. Most studies have addressed the impact on the aquatic environment, as water is an important source of anthropogenic pollutants. Few investigations have been conducted on terrestrial animals living near treatment ponds. We isolated extended-spectrum-β-lactamase Enterobacter cloacae complex-producing strains from 35 clinical isolates, 29 samples of wastewater, 19 wild animals, and 10 domestic animals living in the hospital sewers and at or near a wastewater treatment plant to study the dissemination of clinically relevant resistance through hospital and urban effluents. After comparison of the antibiotic-resistant profiles of E. cloacae complex strains, a more detailed analysis of 41 whole-genome-sequenced strains demonstrated that the most common sequence type, ST114 (*n* = 20), was present in human (*n* = 9) and nonhuman (*n* = 11) samples, with a close genetic relatedness. Whole-genome sequencing confirmed local circulation of this pathogenic lineage in diverse animal species. In addition, nanopore sequencing and specific synteny of an IncHI2/ST1/*bla*_CTX-M-15_ plasmid recovered on the majority of these ST114 clones (*n* = 18) indicated successful worldwide diffusion of this mobile genetic element.

## Abstract (precis)

Wastewater treatment plants are considered hot spots for antibiotic resistance. Most studies have addressed the impact on the aquatic environment, as water is an important source of anthropogenic pollutants.

## INTRODUCTION

Enterobacter cloacae complex (ECC) is widely distributed and has been isolated from many sources (1). Its members are leading causes of nosocomial and opportunistic human infections in clinical settings (2, 3). Unfortunately, in the past decade, treatment and environmental self-care strategies against this bacterial complex have been limited by the emergence of multidrug-resistant isolates and acquired resistance, such as to extended-spectrum-β-lactamase (ESBL); carbapenemase production is of particular concern (4–6). Accurate identification of ECC members is therefore important to better understand ECC’s global epidemiology (3). Various approaches have been used to classify ECC members, most often by sequence analysis of certain housekeeping genes, especially the partial *hsp60* sequence, which permits classification of this complex into 14 genetic clusters (7, 8). Detailed differentiation into 22 genomospecies (A to V) is provided by whole-genome sequencing (WGS) (9, 10). Other recent taxonomic updates illustrate the complexity of this genus and the difficulty of correct assignment (11). Strains belonging to *hsp60* genetic clusters III, VI, and VIII have been found to predominate in human infections (7, 12, 13). Moreover, several studies have reported the worldwide emergence and spread of multidrug-resistant clones in hospital and community settings (5, 14) and also in veterinary medicine (15, 16).

Antibiotic resistance is not restricted to human and veterinary medicine but also affects the environment and wild fauna, with some critical interfaces with human activities, including human sewage and its treatment (17). Wastewater concentrates highly diverse resistant and pathogenic bacterial lineages, in addition to components that maintain selective pressure and a high rate of resistance (18). Treatment processes do not remove these pollutants entirely, and they thus affect the aquatic environment and the microbiome composition of nearby wild fauna (19–21).

In Guadeloupe, a French overseas territory in the Caribbean with extensive resources (22), few recent data are available on resistance to antibiotics. In 2018, *Enterobacteriaceae* were the leading cause of hospital-acquired infection at the University Hospital of Guadeloupe (UHG), with Escherichia coli (15.9% of all infections) and Klebsiella pneumoniae complex species (13.3%) predominating. ESBL-producing isolates were found in 33.8% of the K. pneumoniae complex and only 5.0% of E. coli isolates. ECC infections were less frequent (4.3%), but 19.7% of the isolates were ESBL producers (S. Breurec, personal communication).

Local wastewater in Guadeloupe contained ESBL-producing *Enterobacteriaceae*, including ECC strains (21), but no data were available on possible dissemination of specific ECC clones involved in human health and carrying ESBL determinants. A preliminary study showed no ESBL-producing ECC strains in wild fauna in contrast to other bacterial species (23).

The main objective of this “one health” study was to assess the diverse ESBL-producing ECC strains and the role of wastewater treatment plants (WWTPs) in disseminating specific multidrug-resistant isolates from human sources to animals living near the plants. Indeed, because of the absence of ESBL-producing ECC in a nonpolluted environment, the dissemination of multidrug-resistant isolates or of resistance genes from human ECC isolates to this environment was probable.

## RESULTS

### ESBL-producing ECC lineage in an area with high human activity.

ESBL-producing ECC strains were found in 29 of the 119 samples (24.4%) from animals in all classes screened. The prevalence of positive samples was higher in chickens (8/11, 72.7%) than in other domestic animals (2/21, 9.5%) or in wild fauna (19/87, 21.8%). ESBL-producing ECC strains were also isolated from 29 wastewater samples (74.4%) at the 4 sampling sites (see Table S1). Of the 139 unique ESBL-producing ECC strains, 63 were collected from raw water carried from the hospital sewers to the WWTP effluents, 10 were collected from domestic animals, and 30 were collected from wild fauna (Table 1). Analysis of the antibiotic resistance profiles (ARPs) of these 139 strains supplemented with ARPs from 36 clinical strains indicated that one corresponding to ESBL-producing ECC strains harboring coresistance to fluoroquinolones, gentamicin, and trimethoprim-sulfamethoxazole predominated and was the only one common to the different sample types (ARP–1, 63/139) (Table 1 and Data Set S1). Thirty-one strains with this ARP were selected for WGS. No other ARPs were available from domestic animals; however, to reflect the initial diversity of clinical samples and to explore a putative association between the UHG, the WWTP, and the nearby environment, 10 other clinical ECC strains with 6 ARPs (2, 3, 4, 6, 12, and 14) were also sequenced (see Data Set S1).

**TABLE 1:** Antibiotic-resistant profiles (ARPs) of E. cloacae complex strains (*n* = 139) isolated from humans, wastewater (WW), and wild or domestic animals^*a*^

Antibiotic resistant profile	No. of associated strains	Strain distribution							Resistance details						
		Human	WW site 1	WW site 2	WW site 3	WW site 4	Wild fauna	Domestic animal	Ertapenem	Nalidixic acid	Ciprofloxacin	Gentamicin	Amikacin	Tigecycline	Trimethoprime/sulfamethoxazole
	[*N* (%)]	(*N* = 36)	(*N* = 27)	(*N* = 9)	(*N* = 13)	(*N* = 14)	(*N* = 30)	(*N* = 10)							
ARP–1	63 (45.3)	20	8	1	4	6	14	10	s	R	R	R	s	s	R
ARP–2	20 (14.4)	2	9	1	3		5		s	R	R	R	s	s	s
ARP–3	15 (10.8)	4	1	3	2	1	4		s	R	R	s	s	s	R
ARP–4	6 (4.3)	3			1		2		R	R	R	R	s	s	R
ARP–5	5 (3.6)		1	2		1	1		s	s	s	s	s	s	R
ARP–6	5 (3.6)	2	1			1	1		R	R	R	s	s	s	R
ARP–7	4 (2.9)		1		1	2			R	R	R	R	s	s	s
ARP–8	3 (2.2)		2	1					s	s	s	s	s	s	s
ARP–9	3 (2.2)		1			1	1		s	s	s	R	s	s	R
ARP–10	3 (2.2)		1	1		1			s	R	R	s	s	s	s
ARP–11	2 (1.4)		2						s	s	s	R	s	s	s
ARP–12	2 (1.4)	2							s	R	R	R	s	R	R
ARP–13	2 (1.4)				1		1		s	R	R	R	R	s	s
ARP–14	2 (1.4)	2							R	R	R	R	s	R	R
ARP–15	1 (0.7)	1							s	R	s	R	s	s	R
ARP–16	1 (0.7)				1				R	R	s	s	s	s	s
ARP–17	1 (0.7)						1		s	R	R	R	R	R	s
ARP–18	1 (0.7)					1			R	R	R	s	s	s	s

a All of the 18 ARPs obtained against the 13 antibiotics were unique and associated with strains from different sample types. Intermediate or resistant results are classified as “R,” while “s” corresponds to susceptible phenotypes. All analyzed strains were classified as “R” to ampicillin, amoxicillin–clavulanic acid, cefepime, cefoxitin, cefotaxime, and ceftazidime.

Five different genomospecies were identified among the sequenced ECC strains. Only 3 of these clades defined by Sutton and colleagues were found in human and nonhuman samples (9, 10). ECC clade A, currently named Enterobacter hormaechei subsp. *xiangfangensis*, was the most prevalent, with 15 strains in clinical samples and 11 in other sample types. The second most prevalent clade was ECC clade B (E. hormaechei subsp. *steigerwaltii*), which was found mainly in human samples (7/9). The last shared clade was ECC clade L (*n* = 4) but was found only in human and wastewater isolates (Table 2 and Data Set S1). Overall, the sequence types (STs) showed little heterogeneity, with only two STs (ST113 and ST114) representing 58.5% of the sequenced strains. In nonhuman isolates, ST114 (*dnaA*: 53; *fusA*: 35; *gyrB*: 20; *leuS*: 44; *pyrG*: 45; *rplB*: 4; *rpoB*: 6) was the most common (11/17) and belonged to ECC clade A. Among the six other nonhuman strains in our collection, we reported strains that belonged to clade L (ST598; *n* = 3) and two new STs. The complete profiles of the 7 housekeeping genes used in the ECC multilocus sequence typing scheme (MLST) are presented in Data Set S1. Greater diversity was found among the clinical isolates, with four different STs clearly identified in the ECC clade A, including 9 strains belonging to the predominant ST114 (Table 2 and Data Set S1) and three different STs identified in clinical ECC clade B, 4 of 7 belonging to ST113 (*dnaA*: 4; *fusA*: 22; *gyrB*: 68; *leuS*: 69; *pyrG*: 37; *rplB*: 4; *rpoB*: 24).

**TABLE 2:** Identification of sequence type (ST) and E. cloacae complex (ECC) by sample origin

Current species or subspecies names (8–10)	ECC clade and associated *hsp60* cluster	No. of strains collected	Origin				Identified ST	
			Human	Domestic animal	Wild fauna	Wastewater	Human isolates	Nonhuman isolates
		(*N* = 41)	(*N* = 24)	(*N* = 4)	(*N* = 6)	(*N* = 7)		
E. hormaechei subsp. *xiangfangensis*	A (VI)	26	15	4	4	3	114, 171, 544, 1468^*a*^, na^*b*^	114
E. hormaechei subsp. *steigerwaltii*	B (VIII)	9	7		1	1	113, 133, 190	1474^*a*^
E. cloacae complex clade L	L (−)	4	1			3	1503^*a*^	598
E. cloacae complex clade N	N (−)	1			1			1505^*a*^
E. cloacae complex clade S	S (XIV)	1	1				873	

a New STs; details of target sequences are given in Data Set S1.

b na, ST not attributed.

### Phylogenetic and temporal analyses of ECC ST114 genomes.

**FIG 1:**
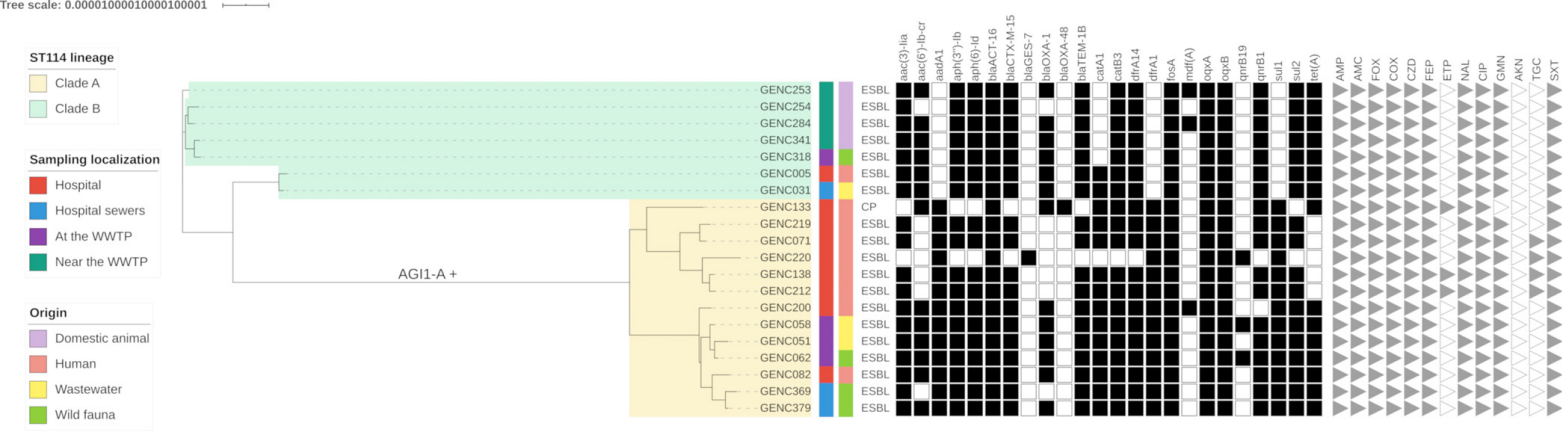
Maximum likelihood phylogenetic tree of E. cloacae complex ST114 isolates from Guadeloupe. Hosts and sampling locations, from the hospital to the wastewater treatment plant (WWTP), are indicated by vertical colored strips. The tree is subdivided into two lineages, ST114-A (in yellow) and ST114-B (in green). All isolates except one carbapenemase-producing strain (CP) are extended-spectrum-β-lactamase (ESBL) producers. Antibiotic resistance genes characterized by ResFinder are indicated by black squares (Data Set S1). Antimicrobial resistance profiles are indicated by gray triangles. AMP, ampicillin; MC, amoxicillin–clavulanic acid: FOX, cefoxitin; COX, cefotaxime; CZD, ceftazidime; FEP, cefepime; ETP, ertapenem; NAL, nalidixic acid; CIP, ciprofloxacin; GMN, gentamicin; AKN, amikacin; TGC, tigecycline; SXT, trimethoprim-sulfamethoxazole. The branch of the genomic island AGI1-A is indicated.

As noted above, ST114 was the only ST detected in all the samples collected, i.e., in 9 from humans, 3 from wastewater, 4 from wild animals, and 4 from domestic animals (see Data Set S1). Maximum likelihood phylogenetic analysis after recombinant removal and based on 814 single-nucleotide polymorphisms (SNPs) revealed clear division of the ST114 isolates into 2 main clusters, A and B (Fig. 1). The bacterial isolates were shown to be ESBL producers (*bla*_CTX-M-15_ and *bla*_GES-7_), except for one that harbored a carbapenemase determinant (*bla*_OXA-48_). The ST114-A lineage consisted mainly of ECC sampled at the hospital (*n* = 8), with 3 strains isolated from wild fauna and 2 from wastewater. Two positive cockroaches (GENC369 and 379) were caught in the UHG sewers (site 1), and anoles were sampled at the WWTP. Cluster ST114-B corresponded mainly to isolates collected near the WWTP from 4 domestic animals, while 1 strain was sampled from a patient, another strain was sampled in the UHG sewer, and 1 strain was sampled from a wild bird at the WWTP. Nucleotidic diversity was higher for the ST114-A lineage (153 mean single-nucleotide polymorphisms (SNPs), minimum 28, maximum 280) than for ST114-B (60 mean SNPs, minimum 14, maximum 100). In the ST114-B lineage, strains GENC318 and GENC341 differed by only 14 SNPs, and strains GENC005 and GENC031 differed by 17 SNPs.

Using a relaxed clock model in BactDating software and incorporating branch-specific recombination events, we accurately reconstructed and dated the maximum likelihood phylogeny of ECC ST114 isolates (see Fig. S1 and S2). This approach revealed a strong temporal signal in this local data set, with a root-to-tip R^2^ of 0.5, indicating that the model explained half of the variation of the data around its mean; furthermore, a Murray permutation test gave a significant *P* value of 0.015, indicating a strong temporal signal in the data set. After 10^7^ Markov chain Monte Carlo iterations of the model, the effective sample size was greater than 300 for the mean mutation rate μ, with a standard deviation of the per-branch substitution rates σ and coalescent time unit α, and μ is equal to 1.32e+01 (95% credible interval [4.77e+00; 2.45e+01]; σ = 4.39e+00 [0.00e+00; 1.21e+01]; α = 1.32e+01 [4.61e+00; 3.69e+01]) (see Fig. S1 and S2). Posterior means were estimated for the dates of all ancestral nodes, and the most recent common ancestor of these sublineages was dated to 1990 (confidence interval: 1947; 2007). We also estimated the dates of the ancestral nodes for sublineages ST114-A and ST114-B and found an older subdivision for ST114-A (2010 [1994; 2014]) than for ST114-B (2015 [2008; 2017]).

### Genetic background of ESBL-producing ECC ST114 isolates: antibiotic resistance genes, IncHI2/ST1 plasmids, and genomic island AGI1-A.

Three β-lactamase-encoding genes, *bla*_CTX-M-15_ (18/20), *bla*_OXA-1_ (13/20), and *bla*_TEM-1B_ (18/20), were identified in most ST114 isolates, in addition to the chromosomal cephalosporinase (*bla*_ACT-16_) (Fig. 1). Other resistance genes also well represented in most samples were *aac(3)-IIa* (18/20), *aac(6′)-Ib-cr* (13/20), *aph(3′')-Ib* [*strA*] (18/20), *aph(6)-Id* [*strB*] (18/20), *catA1* (14/20), *catB3* (19/20), *dfrA1* (13/20), *dfrA14* (19/20), *qnrB1* (18/20), *sul1* (13/20), *sul2* (18/20), *tet*(A) (15/20), and *aadA1* [*ant(3′')-Ia*] (13/20). In addition, all strains related to the ST114-A lineage had an S83L like mutation on *gyrA* associated with fluoroquinolones resistance in E. coli (see Data Set S1).

**FIG 2:**
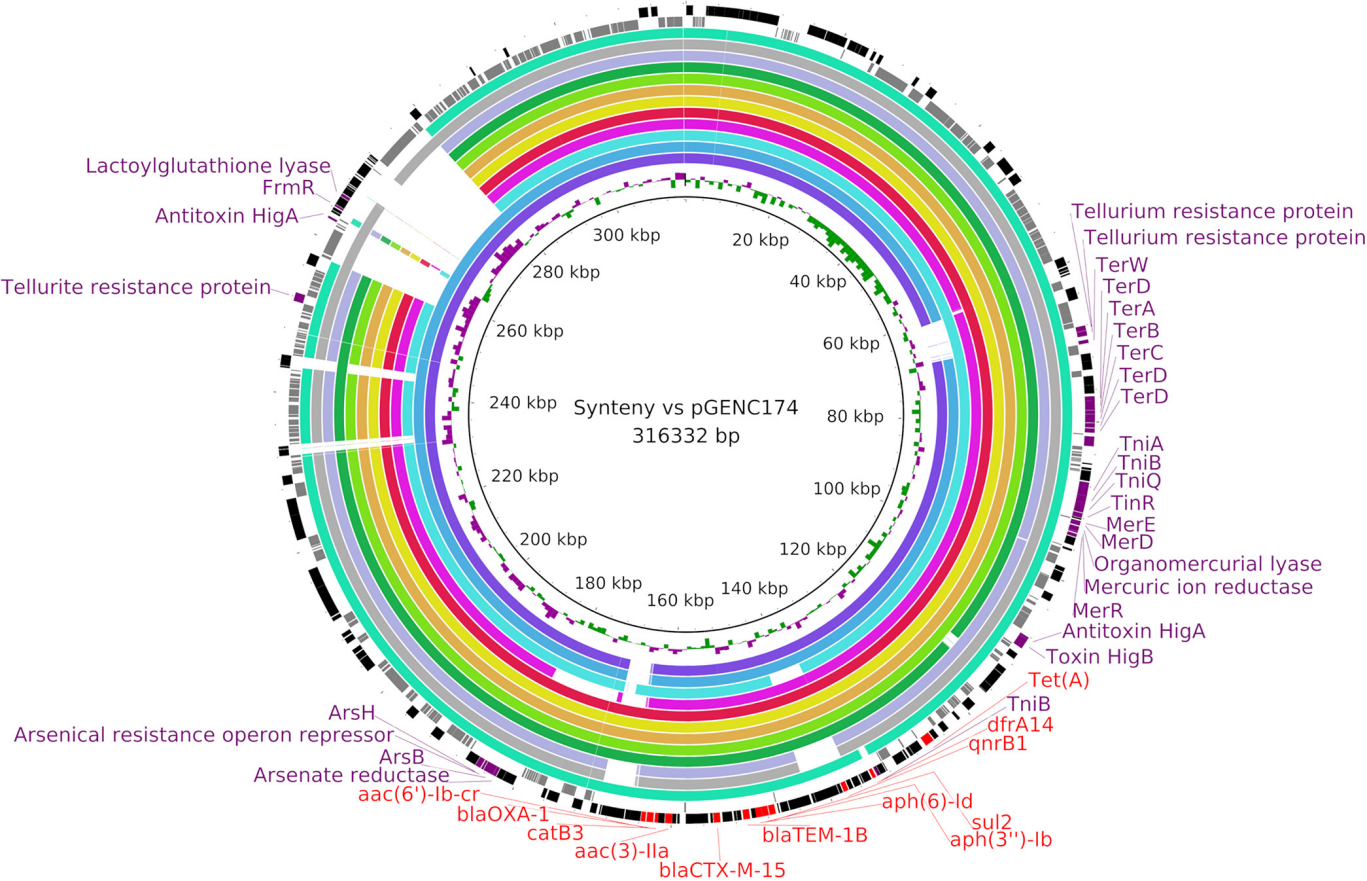
Synteny of the 4 IncHI2/ST1 plasmids obtained from E. cloacae complex (ECC) isolates in Guadeloupe versus 9 reference plasmids from the PLSDB database. Syntenic analysis was performed with BRIG software. The innermost black ring 1 represents the local pGENC174 plasmid (GenBank accession number CP061496, recovered from a rat) associated with its GC skew. The other rings from the inner to the outer represent pairwise comparisons with the following local plasmids: pGENC253 (CP061494, goose), pGENC284 (CP061493, pig), pGENC200 (CP061495, human). Then, the subsequent rings corresponded to close plasmids from the PLSDB database: p23_A-OXA140 (GenBank accession number CP048350, bacterial species Raoultella ornithinolytica, Country Switzerland), pCRENT-193_1 (CP024813, ECC, Republic of Korea), pEB247 (LN830952, ECC, United Republic of Tanzania), pEclC2185CTXM15 (MK736669, ECC, France), pIV_IncHI2_CTX_M_15 (MN540571, Escherichia coli, Italy), pSTm-A54650 (NC_024983, Salmonella enterica, Malawi), pKST313 (LN794248, S. enterica, Kenya), pUGA14_1 (CP021463, S. enterica, Kenya), and pEc21617-310 (MG878867, E. coli, Taiwan). These nine international plasmids were isolated from human samples between 2003 and 2019, except for pIV_IncHI2_CTX_M_15 and p23_A-OXA140 (dog and river, respectively). The last 2 outer rings represent a map of hypothetical proteins (gray boxes) and coding sequences (black boxes); antibiotic resistance genes are indicated by red boxes, and other relevant genes are in purple boxes. Details of the plasmids and complete annotation are provided in Data Set S2.

As most of the ESBL-producing ECC ST114 isolates presented an IncHI2/ST1 plasmidic signature in PlasmidFinder and pMLST (see Fig. S3), we performed ONT sequencing for reconstruction by hybrid assemblage with Illumina full plasmidic sequences of 3 ST114 isolates, the clinical strain GENC200, GENC253 isolated from a goose, and GENC284 isolated from a pig close to the WWTP that collects wastewater from the UHG. For comparison, we added the C-VIII ECC strain GENC174 (ST1474), which has a similar ARP and the same plasmidic signature (see Data Set S1), isolated from a rat at the WWTP. The corresponding plasmids were pGENC200 (291,493 bp), pGENC253 (306,188 bp), pGENC284 (304,958 bp), and pGENC174 (316,332 bp), which presented more than 99.9% nucleotidic identity (Fig. 2). A search for IncHI2/ST1 plasmids in the PLSDB database to match our 4 plasmids resulted in nine additional plasmids that shared more than 88% of the pGENC174 sequence, with more than 99.7% nucleotidic identity. The oldest one was obtained in 2003 from a human sample in Kenya. The corresponding bacterial species were ECC (*n* = 3), Salmonella enterica (*n* = 3), E. coli (*n* = 2), and Raoultella ornithinolytica (*n* = 1) (see Data Set S2). All the plasmids except 2 were from human samples. Determination of synteny in the 13 plasmids by BRIG (Fig. 2) and with Mauve software (see Fig. S4) revealed strong similarity in backbone organization dedicated to replication, stability, leading, and conjugative transfer. All the plasmids had the *bla*_CTX-M-15_, *bla*_OXA-1_, and *bla*_TEM-1B_ encoding genes and also other antibiotic resistance genes, with the presence of *aac(3)-IIa*, *aac(6′)-Ib-cr*, *aadA1*, *aph(3″)-Ib*, *aph(6)-Id*, *catA1*, *catB3*, *dfrA14*, *qnrB1*, *sul2*, and *tet*(A) in most of sequenced plasmids. Resistance to the heavy metals tellurite (*ter* genes), mercury (*mer* and *tni* genes), and arsenic (*ars* genes) was noted in all plasmids; furthermore, resistance to formaldehyde (*frmR* gene) was identified in plasmids from Guadeloupe and from only 1 sample from Kenya (plasmid pUGA14_1). A toxin/antitoxin system was also characterized (*higB*/*higA* genes).

Screening for the genomic resistance island AGI1-A (44,369 bp), previously described in an ECC clinical strain EclC2185 from France (2), identified the complete sequence of AGI1-A (44,358 bp; more than 99.97% coverage versus the reference; 100% nucleotidic identity) in the GENC200 strain. Moreover, a mapping approach to reconstructed AGI1-A in GENC200 made it possible to assemble an identical genomic island in all the ESBL-producing ST114 isolates belonging to lineage ST114-A (*n* = 13), described above (see Table S2), while AGI1-A was absent from the second lineage ST114-B (*n* = 7) (Fig. 1) and other sequenced strains (*n* = 22). Three antibiotic resistance genes were identified on this genomic island with ResFinder: *aadA1*, *dfrA1*, and *sul1*.

## DISCUSSION

### Dissemination of ESBL-producing ECC from hospital to WWTP.

This “one health” study of the spread of ESBL-ECC producers in Guadeloupe from a hospital to the environment near the WWTP demonstrated the carriage of one antibiotic-resistant *Enterobacteriaceae* genus through 19/87 (21.8%) wild fauna living at the WWTP and near the UHG sewer. Previous work on ESBL-producing strains in wildlife in Guadeloupe was less worrisome. Results were focused on E. coli carriage and showed that only 7/884 animals (0.79%) were positive, comprising 5 rats (Rattus norvegicus), 1 Carib grackle (Quiscalus lugubris), and 1 iguana (Iguana iguana) (22). The difference from our results may be due to lower exposure to anthropogenic pollution in the previous work. We sampled numerous wild animals in direct contact with human sewage and waste, including cockroaches caught in sewers, toads in clarifier settling tanks, and rats, anoles, and wild birds near solid waste from the screening and grit removal steps. Our observations corroborate previous observations that wastewater effluents contribute to the spread of human‐associated bacteria and antibiotic resistance determinants through wild fauna (19, 20).

Direct access to the WWTP park did not, however, explain the carriage of determinants in domestic animals (10/32, 31.5%), especially chickens. We propose 2 hypotheses to explain the high carriage of resistance in domestic animals within a radius of more than 500 m of the WWTP. The pigs, chicken, geese, and dogs carrying ESBL strains in our study lived close together in different pens, and the ST114 genetic background assigned was only to the B lineage, including a strain from a Carib grackle (GENC318), a species frequently observed in the area, especially during feeding time. This isolate, recovered in February 2019, differed by only 14 SNPs from GENC341 isolated in March 2019 from a chicken, indicating a close genetic relationship (Fig. 1 and Data Set S1). These observations point to a potential role of wild fauna, especially birds, in the spread of antibiotic-resistant bacteria, as previously described (24). Domestic animal contamination could, however, be older and maintained for several months or years (GENC253, 254, and 284) (Fig. 1 and Data Set S1). In view of the layout of the WWTP and the distance from domestic animal pens (300 m), however, a second hypothesis is dissemination of the lineage by air, resulting in contamination of the environment, such as soil in pens. As some processes in screening and the aeration tank at the WWTP are more likely to create bio-aerosols, bacteria could be emitted outside the treatment plant (25).

Most of the ESBL-producing ECC strains in nonhuman isolates belonged to the ST114 lineage (11/17) (see Data Set S1), mirroring the prevalence in human clinical isolates (9/24). Few studies have been published on ST diversity in clinical ECC; however, ST114, ST78, ST108, and ST66 are the most common in human infections (5, 14). ST114 was described by Girlich et al. as the most prevalent in a worldwide collection of *bla*_CTX-M-15_-producing ECC of clinical origin (26) and has been reported in a hospital outbreak (2). This high-risk ECC ST has been isolated only rarely from domestic animals (15, 16) and only twice previously in wildlife exposed to human activities (27, 28). Like other ESBL-ECC producers, the ST114 clone was also found after wastewater treatment (site 4, GENC051) (see Data Set S1), indicating that pathogenic, highly resistant clones are not totally eliminated during wastewater treatment. Therefore, even after dilution in the sea, they could affect the coastal environment and local marine life (21, 27).

WGS also confirmed the presence of carbapenemase-producing ECC (GENC133) in Guadeloupe (Fig. 1), where carbapenemase-producing *Enterobacteriaceae* emerged in 2014 (29, 30). The isolate collected from a human sample in September 2018 belonged to ST114, and its genetic background was shown to be the major ECC clade A clone in a previous study of carbapenemase producers (5). This finding is of great concern because of the many ST114 clones isolated in clinical samples and because of the capacity of ST114 to spread. No carbapenemase producer has yet been isolated from a nonhuman sample in Guadeloupe (21–23), but this should be monitored in the future.

### Local ESBL-producing ECC ST114 is clearly structured into two distinct lineages.

A maximum likelihood phylogenetic tree of 19 ESBL-producing isolates and 1 carbapenemase producer ECC ST114 isolate revealed a clear division into 2 lineages, ST114-A and ST114-B (Fig. 1). The first comprised 8 clinical strains and 5 isolates from the WWTP (2 samples from wastewater and 3 from animals), and the second comprised 5 strains from animals, 2 strains from wastewater, and 1 human isolate, indicating that clinical and nonclinical ESBL strains are mixed in the same lineage and human strains are transmitted to animals through wastewater. Bayesian analysis in BactDating software (integrating recombinations) separated these 2 lineages and dated the most recent common ancestor to around 1990 (95% credible interval [1947; 2007]) (see Fig. S1 and S2). The nucleotidic diversity of ST114-A (153 mean SNPs, minimum 28, maximum 280) is higher than that of ST114-B (60 mean SNPs, minimum 14, maximum 100). Moreover, ST114-A is probably more ancient than ST114-B (2010 versus 2015). A previous study highlighted a subdivision of ST114 isolates into 4 sublineages (5), but only 1 (strains from Serbia, Romania, and Tunisia), characterized by *bla*_TEM-1_, *bla*_OXA-1_, and *bla*_CTX-M-15_ resistance genes and IncHI2/ST1 plasmids, could be compared to ST114 isolates from Guadeloupe.

Interestingly, the genomic resistance island AGI1-A (44,369 bp) was recovered only from lineage ST114-A (see Table S2). This genomic island was previously described in an ESBL-producing ECC ST114 strain (EclC2185) involved in a nosocomial outbreak in a French hospital and harbors the IncHI2/ST1 plasmid and the same antibiotic resistance genes (Fig. 2). The genomic island, first characterized in Acinetobacter baumannii (2), is a variant of an AGI1 resistance island described in *Salmonella* and *Proteus* and codes for multiple types of antibiotic resistance through the *aadA1*, *dfrA1*, and *sul1* genes. A recent study confirmed that this genomic island was mobilizable (31). Our finding of this integrative mobilizable element in Guadeloupe confirms the high risk of interspecies dissemination of AGI1 worldwide.

The presence of two contrasted sublineages of local ECC ST114 is confirmed by (i) the separation of ST114 isolates into 2 branches, (ii) the finding that more ST114-A isolates are clinical isolates and more ST114-B isolates are nonclinical isolates, (iii) the findings that the subdivision of ST114-A is older than that of ST114-B and ST114-A has greater nucleotidic diversity, and (iv) the finding that only ST114-A has the genomic island AGI1-A and specific mutation in *gyrA*.

### A highly successful IncHI2/ST1 plasmid is circulating in Guadeloupe.

*bla*_CTX-M-15_ was the most common gene coding for ESBL, as described in clinical and veterinary studies on ECC (3, 14–16). This and most of the antimicrobial resistance genes identified were present in local ESBL-ST114 isolates on large conjugative IncHI2/ST1 plasmids (Fig. 2, Fig. S4, and Data Set S1), representing circulation of the same panel of antibiotic resistance genes among isolates of different origins. Synteny analysis showed that these plasmids are closely related to 9 plasmids that were isolated from various bacterial species worldwide and recovered between 2003 and 2019. In addition, the presence of closely related plasmids was suspected by mapping in a study in S. enterica isolates from the Central African Republic, Mali, and Senegal (32). Although this plasmid family has been described mainly in Africa (33), Asia, and Europe, to our knowledge, this is the first description in clinical isolates in the Americas. A similar plasmid has been identified in ESBL-producing E. coli strains recovered from the same wastewater and animal collection and is still under investigation [unpublished data].

This plasmid also has numerous genes encoding resistance to heavy-metal ions: tellurite (*ter*), mercury (*mer* and *tni*), and arsenic (*ars*). A gene related to resistance to formaldehyde (*frmR*) has been identified only in plasmids in Guadeloupe and in S. enterica in Kenya (plasmid pUGA14_1). These genes are probably a key factor in the persistence, selection, and spread of antibiotic-resistant bacteria in hostile environments where they encounter toxic organic substances such as detergents, heavy metals, and other antimicrobials (32). A toxin/antitoxin system has been characterized (*higB*/*higA* genes) that allows persistence of plasmid in bacteria. A lactoylglutathione lyase (also called glyoxalase) has been identified in plasmids of ECC in Guadeloupe which has been described as a virulence factor in view of its critical role in methylglyoxal detoxification (34). Methylglyoxal is produced and secreted by intestinal flora (35) and is responsible for strong oxidative stress; therefore, resistance to such compounds increases the survival of enteric pathogens.

### Conclusion.

Our results, focusing on Enterobacter cloacae complex, confirm that the environment near WWTPs is a hot spot for the emergence of antibiotic-resistant bacteria and their spread to wild fauna that live and feed there. A well-conserved IncHI2/ST1/*bla*_CTX-M-15_ plasmid has spread among ECC strains of different origin (humans, wild fauna, domestic animals, and urban sewage) in Guadeloupe. It is also present in other *Enterobacteriaceae* from different animal and human sources over a wide geographic area, indicating the importance of surveillance programs with plasmid sequencing in limiting its spread.

## MATERIALS AND METHODS

### ESBL-producing ECC isolates from wastewater.

The WWTP studied, the largest in Guadeloupe, located in Jarry, treats wastewater from the UHG and also urban wastewater from three major cities (Pointe-à-Pitre, Les Abymes, and Baie-Mahault). It treats an average influent of 13,904 m^3^/day, for a maximum of 33,051 inhabitant equivalents in 2018. It is an activated sludge process, and the effluent treatment pond is located in an open environment in an industrial area. Effluent is discharged into the sea near Petit Cul-de-Sac Marin.

During 10 sampling campaigns between April 2018 and March 2019, 4 sampling sites were set up along the wastewater continuum between the UHG and the WWTP: samples of untreated clinical sewage outflow at the UHG (Pointe-à-Pitre) (site 1), samples of mixed clinical and urban untreated sewage between the UHG and the WWTP (site 2), and samples from influents and effluents at the WWTP (sites 3 and 4, respectively). Site 2 is located about 2 km from the hospital (site 1), while the WWTP (sites 3 and 4) is about 8 km from the first sampling point. A total of 39 sewage samples were collected during the study period (1 sample from site 2 was missing).

Raw water samples (500 ml) were collected within a time interval of up to 24 h at the different sites, stored at 4°C, and taken to the laboratory, where they were filtered within 4 h, as described previously (21). Membranes were placed on CCA agar plates (CHROMagar, Paris, France) supplemented with ceftriaxone at 4 mg/liter and on a CCA plate without antibiotic as control. Plates were incubated at 37°C for a maximum of 24 h. According to their morphology and the manufacturer’s instructions, 5 colonies presumed to be resistant to third-generation cephalosporins ECC were isolated per sample, if available.

### ESBL-producing ECC isolates from animals living near the wastewater pathway.

Between June 2018 and April 2019, in parallel to the raw water sampling campaigns, wild animals were trapped and sampled at various locations in the WWTP park. Anoles (Anolis marmoratus speciosus) and cane toads (Rhinella marina) were released, while rats (Rattus rattus) were euthanized. A single cloacal swab was taken from 20 anoles, 19 cane toads, and 8 rats, and fresh feces were recovered from 11 Carib grackles (Quiscalus lugubris). We also investigated 32 domestic animals living in a fenced-in park 300 m from the WWTP. A single cloacal or rectal swab was taken from 11 chickens, 10 pigs, and 2 geese, and fresh stools were collected nearby from 6 dogs (*Fila brasileiro*) and 3 cows. None of these domestic animals received antibiotic treatment during the study period and 6 months before the start of sampling. Finally, 29 cockroaches (Periplaneta americana) were trapped in the UHG sewers (site 1, *n* = 20) and the WWTP (*n* = 9), and their alimentary tracts were sampled as described previously (36). The protocol was approved by the Committee for Ethics in animal experiments of the French West Indies and Guyana (reference 971-2016-12-20-001).

All fecal samples were placed at 4°C and processed for enrichment in 9 ml buffered peptone water on the same day. Samples were incubated for 16 to 20 h at 37°C, and then 0.1-ml aliquots were inoculated onto CCA agar supplemented with ceftriaxone at 4 mg/liter and onto a CCA agar plate as control.

### ECC identification and susceptibility analysis.

The species of all isolates were identified by matrix-assisted laser desorption ionization–time of flight mass spectrometry (MALDI-TOF MS) (Shimadzu Biotech, Kyoto, Japan). Susceptibility to ampicillin (10 μg), nalidixic acid (30 μg), amikacin (30 μg), amoxicillin-clavulanic acid (10 μg), cefepime (30 μg), cefotaxime (5 μg), cefoxitin (30 μg), ceftazidime (10 μg), ciprofloxacin (5 μg), ertapenem (10 μg), gentamicin (10 μg), tigecycline (15 μg), and trimethoprim-sulfamethoxazole (1.25 to 23.75 μg) was determined by the disk diffusion method on Mueller-Hinton agar (Bio-Rad). Isolates were classified as resistant, intermediate, or susceptible according to the 2018 guidelines of CA-SFM/EUCAST (http://www.sfm-microbiologie.org), and production of ESBL was detected by the double-disk synergy test on Mueller-Hinton agar with cloxacillin (in case of cephalosporinase overproduction) or without, according to CA-SFM/EUCAST recommendations. Isolates of intermediate susceptibility were combined with resistant isolates for data analysis. If more than 1 isolate with the same ARP was recovered from the same sample, only the first was retained for analysis. All isolates, independently of their origin, were classified according to their ARP against the 13 antibiotics. Each profile represented resistance to a unique composition of antibiotics selected for this study.

In a one health perspective, we also added 36 ARPs from ESBL-producing ECC isolates associated with human infections at the UHG, recovered in 2018, and analyzed as described in the previous paragraph. The ARPs of all the bacteria of human origin were similar to those in previous environmental samples and were characterized as ESBL or carbapenemase producers. If multiple strains were obtained from a patient, only 1 was kept. The protocol for collecting bacteria was in accordance with the requirements of the local ethics committee (reference A5_19_12_05_TRAMID).

### WGS and phylogenetic analyses.

A selection of ESBL-producing ECC strains was done by focusing on predominant ARPs found in animals, wastewater, and humans. The prevalence of different ARPs is shown in Table 1. The 10 strains from animals and the 7 strains from wastewater sampling points selected for WGS were found to have similar profiles against the 13 antibiotics with ARP–1 (Table 1 and Table S1). In addition, 14 human isolates that also clustered in ARP–1 and 10 clinical strains with other ARPs were randomly selected for WGS. Total bacterial DNA was extracted from pure cultures with a Qiagen QIAamp DNA minikit (Qiagen, Hilden, Germany). WGS was performed at the Plateforme de microbiologie mutualisée of the Pasteur International Bioresources network (Institut Pasteur, Paris, France). Libraries were prepared with an Illumina Nextera XT kit and further sequenced with the NextSeq 500 system (paired-end 150 bp). Reads were trimmed and filtered with an AlienTrimmer (37), yielding a mean 96-fold estimated coverage.

Genomes were assembled with SPAdes software v3.9.0 (38). The quality of the assemblies was checked with QUAST software (39), which gave a mean *N*_50_ of 251,153 (minimum 181,264, maximum 388,439). ECC clades were initially identified with the ECC references of Chavda, Sutton, and Beyrouthy (8–10). After identification, MLST was performed *in silico* with MLST software (https://github.com/tseemann/mlst) associated with PubMLST (40). Definitions of new alleles and STs were provided by the same database. The antibiotic resistance gene content was assessed with ResFinder (41), plasmids were identified and typed with PlasmidFinder and pMLST (42), and virulence genes were detected with Abricate software (https://github.com/tseemann/abricate) and VFDB (43). In addition, *gyrA* mutation associated with fluoroquinolones resistance was screened with CARD (44).

The most common ST in this collection, ST114, was analyzed in depth. Total core SNPs were detected with SNIPPY software and GENC200 strain as reference (https://github.com/tseemann/snippy). Recombination sequences were further identified and removed from the global core genome alignment with ClonalFrameML (45), giving a total of 814 SNPs (minimum of 14 SNPs; maximum of 459). Maximum likelihood phylogenetic reconstruction was performed with RAxML v8 (46) in the GTR-CAT model and 1,000 bootstrap replicates, and the tree was drawn with iTOL (47).

### Temporal analyses of ECC ST114 genomes.

The presence of a time signal was first evaluated by calculating the linear regression between the root-to-tip distance and the isolation year (see Fig. S2). Null distributions of R^2^ values were generated by 1,000 permutations on the actual isolation year by random permutations, as described by Murray et al. (48). The ClonalFrameML output was further used as input for BactDating analyses (49) to perform Bayesian dating of the nodes of the ECC ST114 phylogenetic tree in a relaxed clock model, after incorporating information on branch-specific recombination rates. The model was run for 10^7^ iterations to ensure perfect convergence of the Markov chain Monte Carlo and the significance of effective sample size for inferred key parameters, i.e., mean mutation rate μ, standard deviation of the per-branch substitution rates σ, and coalescence time unit α.

### Sequencing and synteny of IncHI2 plasmids.

Previous analyses revealed the presence of IncHI2/ST1 plasmids in most of the ECC ST114 strains. We used nanopore sequencing (Oxford Nanopore Technology [ONT]) to fully sequence corresponding plasmids in ECC strains GENC200, GENC253, and GENC284 sampled from a human, a goose, and a pig, respectively. For comparison, we added the GENC174 strain from our bacterial collection, as this cluster VIII isolate sampled in 2018 from a rat also presented an IncHI2/ST1 plasmidic signature and a close antibiotic resistance profile. ONT libraries were prepared (without shearing steps to select for long reads) with an SQK-LSK109 ligation sequencing kit and an EXP-NBD104 native barcoding expansion 1-12 kit. Genomes were sequenced for 48 h on a FLO-MIN106 flow cell connected to the MinION device. Reads were then base-called externally with Guppy software v3.6.0 and further debarcoded and screened for quality with EPI2ME v2020.2.10 FASTQ barcoding workflow. A mean read size of 6,731 bp (minimum 4,903; maximum 8,437) and mean estimated coverage of 44-fold were obtained. Hybrid assembly was then performed with both high-quality Illumina and nanopore reads in a Unicycler pipeline (50), resulting in a final mean coverage of 135-fold. Assembled, circularized contigs corresponding to IncHI2/ST1 plasmids were identified with PlasmidFinder and pMLST, and antibiotic resistance was confirmed with ResFinder. We recovered full sequences of the targeted plasmids pGENC200, pGENC253, pGENC284, and pGENC174, which were annotated on a RAST server (51). Similar plasmids were then sought with Mash Screen in the plasmids database PLSDB (52), a resource containing 20,668 plasmid sequences in June 2020, collected from the NCBI nucleotide database. Syntenic analysis of all plasmids versus pGENC174 was performed with BRIG and Mauve software (53, 54). Antibiotic resistances genes of selected PLSDB plasmids were explored with ResFinder.

### Screening of genomic island AGI1-A in ECC ST114 strains.

The genomic resistance island AGI1-A was previously described in ECC strain EclC2185 (GenBank accession number MH545561) (2). Hybrid genome assembly on GENC200 was aligned against AGI1-A, and the corresponding sequence was extracted. In order to reconstruct the putative AGI1-A type in our ECC collection, FASTQ reads of ST114 isolates were mapped against an AGI1-A sequence extracted from GENC200 with Bowtie 2 software (55). Selected mapped reads were assembled with SPAdes, and the quality of the assembly was checked with QUAST software against the reference GENC200 strain. Antibiotic resistance genes were identified on assembled genomic island with ResFinder.

### Data availability.

All read sequences for the 41 WGS strains and the 4 plasmids have been deposited in the GenBank database under BioProject numbers PRJNA649757 and PRJNA659514. Corresponding accession numbers are listed in Data Set S1 (strains) and Data Set S2 (plasmids).

## Supplementary Material

Supplemental file 1

Supplemental file 2

Supplemental file 3

## References

[1] Davin-Regli A, Lavigne JP, Pagès JM. 2019. *Enterobacter* spp.: update on taxonomy, clinical aspects, and emerging antimicrobial resistance. Clin Microbiol Rev 32:1–32. doi:10.1128/CMR.00002-19. PMC6750132 31315895

[2] Siebor E, De Curraize C, Neuwirth C. 2019. Identification of AGI1-A, a variant of *Acinetobacter* genomic island 1 (AGI1), in a French clinical isolate belonging to the *Enterobacter cloacae* complex. J Antimicrob Chemother 74:311–314. doi:10.1093/jac/dky442. 30412264

[3] Garinet S, Fihman V, Jacquier H, Corvec S, Le Monnier A, Guillard T, Cattoir V, Zahar JH, Woerther PL, Carbonnelle E, Wargnier A, Kernéis S, Morand PC, GMC. 2018. Elective distribution of resistance to beta-lactams among *Enterobacter cloacae* genetic clusters. J Infect 77:178–182. doi:10.1016/j.jinf.2018.05.005. 29807092

[4] Rice LB. 2008. Federal funding for the study of antimicrobial resistance in nosocomial pathogens: no ESKAPE. J Infect Dis 197:1079–1081. doi:10.1086/533452. 18419525

[5] Peirano G, Matsumura Y, Adams MD, Bradford P, Motyl M, Chen L, Kreiswirth BN, Pitout JDD. 2018. Genomic epidemiology of global carbapenemase-producing *Enterobacter* spp., 2008–2014. Emerg Infect Dis 24:1010–1019. doi:10.3201/eid2406.171648. 29774858 PMC6004858

[6] World Health Organization. 2017. WHO publishes list of bacteria for which new antibiotics are urgently needed. World Health Organization, Geneva, Switzerland. https://www.who.int/news/item/27-02-2017-who-publishes-list-of-bacteria-for-which-new-antibiotics-are-urgently-needed.

[7] Hoffmann H, Roggenkamp A. 2003. Population genetics of the nomenspecies *Enterobacter cloacae*. Appl Environ Microbiol 69:5306–5318. doi:10.1128/aem.69.9.5306-5318.2003. 12957918 PMC194928

[8] Beyrouthy R, Barets M, Marion E, Dananché C, Dauwalder O, Robin F, Gauthier L, Jousset A, Dortet L, Guérin F, Bénet T, Cassier P, Vanhems P, Bonnet R. 2018. Novel *Enterobacter* lineage as leading cause of nosocomial outbreak involving carbapenemase-producing strains. Emerg Infect Dis 24:1505–1515. doi:10.3201/eid2408.180151. 30014838 PMC6056098

[9] Chavda KD, Chen L, Fouts DE, Sutton G, Brinkac L, Jenkins SG, Bonomo RA, Adams MD, Kreiswirth BN. 2016. Comprehensive genome analysis of carbapenemase-producing *Enterobacter* spp.: new insights into phylogeny, population structure, and resistance mechanisms. mBio 7:1–16. doi:10.1128/mBio.02093-16. PMC5156309 27965456

[10] Sutton GG, Brinkac LM, Clarke TH, Fouts DE. 2018. *Enterobacter hormaechei* subsp. *hoffmannii* subsp. nov., *Enterobacter hormaechei* subsp. *xiangfangensis* comb. nov., *Enterobacter roggenkampii* sp. nov., and *Enterobacter muelleri* is a later heterotypic synonym of *Enterobacter asburiae* based on computational analysis of sequenced *Enterobacter* genomes. F1000Res 7:521. doi:10.12688/f1000research.14566.2. 30430006 PMC6097438

[11] Wu W, Feng Y, Zong Z. 2020. Precise species identification for *Enterobacter*: a genome sequence-based study with reporting of two novel species, *Enterobacter quasiroggenkampii* sp. nov. and *Enterobacter quasimori* sp. nov. mSystems 5:e00527-20. doi:10.1128/mSystems.00527-20. 32753511 PMC7406230

[12] Hoffmann H, Stindl S, Ludwig W, Stumpf A, Mehlen A, Monget D, Pierard D, Ziesing S, Heesemann J, Roggenkamp A, Schleifer KH. 2005. *Enterobacter hormaechei* subsp. *oharae* subsp. nov., *E. hormaechei* subsp. *hormaechei* comb. nov., and *E. hormaechei* subsp. *steigerwaltii* subsp. nov., three new subspecies of clinical importance. J Clin Microbiol 43:3297–3303. doi:10.1128/JCM.43.7.3297-3303.2005. 16000451 PMC1169129

[13] Kremer A, Hoffmann H. 2012. Prevalences of the *Enterobacter cloacae* complex and its phylogenetic derivatives in the nosocomial environment. Eur J Clin Microbiol Infect Dis 31:2951–2955. doi:10.1007/s10096-012-1646-2. 22648160

[14] Izdebski R, Baraniak A, Herda M, Fiett J, Bonten MJM, Carmeli Y, Goossens H, Hryniewicz W, Brun-Buisson C, Gniadkowski M, MOSAR WP2, WP3 and WP5 Study Groups. 2015. MLST reveals potentially high-risk international clones of *Enterobacter cloacae*. J Antimicrob Chemother 70:48–56. doi:10.1093/jac/dku359. 25216820

[15] Haenni M, Saras E, Ponsin C, Dahmen S, Petitjean M, Hocquet D, Madec J-Y. 2016. High prevalence of international ESBL CTX-M-15-producing *Enterobacter cloacae* ST114 clone in animals. J Antimicrob Chemother 71:1497–1500. doi:10.1093/jac/dkw006. 26850718

[16] Harada K, Shimizu T, Mukai Y, Kuwajima K, Sato T, Kajino A, Usui M, Tamura Y, Kimura Y, Miyamoto T, Tsuyuki Y, Ohki A, Kataoka Y. 2017. Phenotypic and molecular characterization of antimicrobial resistance in *Enterobacter* spp. isolates from companion animals in Japan. PLoS One 12:e0174178-12. doi:10.1371/journal.pone.0174178. 28328967 PMC5362103

[17] Kraemer SA, Ramachandran A, Perron GG. 2019. Antibiotic pollution in the environment: from microbial ecology to public policy. Microorganisms 7:180. doi:10.3390/microorganisms7060180. PMC6616856 31234491

[18] Gao P, He S, Huang S, Li K, Liu Z, Xue G, Sun W. 2015. Impacts of coexisting antibiotics, antibacterial residues, and heavy metals on the occurrence of erythromycin resistance genes in urban wastewater. Appl Microbiol Biotechnol 99:3971–3980. doi:10.1007/s00253-015-6404-9. 25631280

[19] Marcelino VR, Wille M, Hurt AC, González-Acuña D, Klaassen M, Schlub TE, Eden JS, Shi M, Iredell JR, Sorrel TC, Holmes EC. 2019. Meta-transcriptomics reveals a diverse antibiotic resistance gene pool in avian microbiomes. BMC Biol 17:311–311. doi:10.1186/s12915-019-0649-1. PMC6454771 30961590

[20] Swift BMC, Bennett M, Waller K, Dodd C, Murray A, Gomes RL, Humphreys B, Hobman JL, Jones MA, Whitlock SE, Mitchell LJ, Lennon RJ, Arnold KE. 2019. Anthropogenic environmental drivers of antimicrobial resistance in wildlife. Sci Total Environ 649:12–20. doi:10.1016/j.scitotenv.2018.08.180. 30170212

[21] Guyomard-Rabenirina S, Dartron C, Falord M, Sadikalay S, Ducat C, Richard V, Breurec S, Gros O, Talarmin A. 2017. Resistance to antimicrobial drugs in different surface waters and wastewaters of Guadeloupe. PLoS One 12:e0173155-17. doi:10.1371/journal.pone.0173155. 28253356 PMC5333909

[22] Guyomard-Rabenirina S, Reynaud Y, Pot M, Albina E, Couvin D, Ducat C, Gruel G, Ferdinand S, Legreneur P, Le Hello S, Malpote E, Sadikalay S, Talarmin A, Breurec S. 2020. Antimicrobial resistance in wildlife in Guadeloupe (French West Indies): distribution of a single *bla*_CTX–M–1_/IncI1/ST3 plasmid among humans and wild animals. Front Microbiol 11:1524. doi:10.3389/fmicb.2020.01524. 32754130 PMC7366356

[23] Guyomard-Rabenirina S. 2016. Résistance aux antibiotiques des entérobactéries en Guadeloupe: importance en milieu communautaire et diffusion environnementale. PhD thesis. Université des Antilles, Pointe-à-Pitres, Guadeloupe.

[24] Ahlstrom CA, Bonnedahl J, Woksepp H, Hernandez J, Reed JA, Tibbitts L, Olsen B, Douglas DC, Ramey AM. 2019. Satellite tracking of gulls and genomic characterization of faecal bacteria reveals environmentally mediated acquisition and dispersal of antimicrobial‐resistant *Escherichia coli* on the Kenai Peninsula. Mol Ecol 28:2531–2545. doi:10.1111/mec.15101. 30980689

[25] Li J, Zhou L, Zhang X, Xu C, Dong L, Yao M. 2016. Bioaerosol emissions and detection of airborne antibiotic resistance genes from a wastewater treatment plant. Atmos Environ 124:404–412. doi:10.1016/j.atmosenv.2015.06.030.

[26] Girlich D, Poirel L, Nordmann P. 2015. Clonal distribution of multidrug-resistant *Enterobacter cloacae*. Diagn Microbiol Infect Dis 81:264–268. doi:10.1016/j.diagmicrobio.2015.01.003. 25680336

[27] Goldberg DW, Fernandes MR, Sellera FP, Costa DGC, Loureiro Bracarense AP, Lincopan N. 2019. Genetic background of CTX-M-15-producing *Enterobacter hormaechei* ST114 and *Citrobacter freundii* ST265 co-infecting a free-living green turtle (*Chelonia mydas*). Zoonoses Public Health 66:540–545. doi:10.1111/zph.12572. 30843359

[28] Desvars-Larrive A, Ruppitsch W, Lepuschitz S, Szostak MP, Spergser J, Feßler AT, Schwarz S, Monecke S, Ehricht R, Walzer C, Loncaric I. 2016. Urban brown rats (*Rattus norvegicus*) as possible source of multidrug-resistant *Enterobacteriaceae* and meticillin-resistant *Staphylococcus* spp., Vienna, Austria, 2016 and 2017. Euro Surveill 24:1900149. doi:10.2807/1560-7917.ES.2019.24.32.1900149. PMC6693289 31411133

[29] Bastian S, Nordmann P, Creton E, Malpote E, Thiery G, Martino F, Breurec S, Dortet L. 2015. First case of NDM-1 producing *Klebsiella pneumoniae* in Caribbean islands. Int J Infect Dis 34:53–54. doi:10.1016/j.ijid.2015.03.002. 25747780

[30] Breurec S, Bastian S, Cuzon G, Bernabeu S, Foucan T, Galanth S, Naas T, Dortet L. 2015. Emergence of OXA-48-producing *Escherichia coli* in the Caribbean islands. J Glob Antimicrob Resist 3:217–218. doi:10.1016/j.jgar.2015.04.004. 27873713

[31] Siebor E, Neuwirth C. 2020. New insights regarding *Acinetobacter* genomic island-related elements. Int J Antimicrob Agents 56:106117. doi:10.1016/j.ijantimicag.2020.106117. 32745526

[32] Breurec S, Reynaud Y, Frank T, Farra A, Costilhes G, Weill F-X, Le Hello S. 2019. Serotype distribution and antimicrobial resistance of human *Salmonella enterica* in Bangui, Central African Republic, from 2004 to 2013. PLoS Negl Trop Dis 13:e0007917-13. doi:10.1371/journal.pntd.0007917. 31790418 PMC6907862

[33] Pati NB, Doijad SP, Schultze T, Mannala GK, Yao Y, Jaiswal S, Ryan D, Suar M, Gwozdzinski K, Bunk B, Mraheil MA, Marahiel MA, Hegemann JD, Spröer C, Goesmann A, Falgenhauer L, Hain T, Imirzalioglu C, Mshana SE, Overmann J, Chakraborty T. 2018 *Enterobacter bugandensis*: a novel enterobacterial species associated with severe clinical infection Sci Rep 8:5392. doi:10.1038/s41598-018-23069-z. 29599516 PMC5876403

[34] Chakraborty S, Gogoi M, Chakravortty D. 2015. Lactoylglutathione lyase, a critical enzyme in methylglyoxal detoxification, contributes to survival of *Salmonella* in the nutrient rich environment. Virulence 6:50–65. doi:10.4161/21505594.2014.983791. 25517857 PMC4603430

[35] Baskaran S, Rajan DP, Balasubramanian KA. 1989. Formation of methylglyoxal by bacteria isolated from human faeces. J Med Microbiol 28:211–215. doi:10.1099/00222615-28-3-211. 2926792

[36] Pai H. 2013. Multidrug resistant bacteria isolated from cockroaches in long-term care facilities and nursing homes. Acta Trop 125:18–22. doi:10.1016/j.actatropica.2012.08.016. 22960645

[37] Criscuolo A, Brisse S. 2014. AlienTrimmer removes adapter oligonucleotides with high sensitivity in short-insert paired-end reads. Commentary on Turner (2014) Assessment of insert sizes and adapter content in FASTQ data from NexteraXT libraries. Front Genet 5:130. doi:10.3389/fgene.2014.00130. 24860597 PMC4026695

[38] Bankevich A, Nurk S, Antipov D, Gurevich AA, Dvorkin M, Kulikov AS, Lesin VM, Nikolenko SI, Pham S, Prjibelski AD, Pyshkin AY, Sirotkin AV, Vyahhi N, Tesler G, Alekseyev MA, Pevzner PA. 2012. SPAdes: a new genome assembly algorithm and its applications to single-cell sequencing. J Comput Biol 19:455–477. doi:10.1089/cmb.2012.0021. 22506599 PMC3342519

[39] Gurevich A, Saveliev V, Vyahhi N, Tesler G. 2013. QUAST: quality assessment tool for genome assemblies. Bioinformatics 29:1072–1075. doi:10.1093/bioinformatics/btt086. 23422339 PMC3624806

[40] Jolley KA, Bray JE, Maiden MCJ. 2018. Open-access bacterial population genomics: BIGSdb software, the PubMLST.org website and their applications. Wellcome Open Res 3:124. doi:10.12688/wellcomeopenres.14826.1. 30345391 PMC6192448

[41] Zankari E, Hasman H, Cosentino S, Vestergaard M, Rasmussen S, Lund O, Aarestrup FM, Larsen MV. 2012. Identification of acquired antimicrobial resistance genes. J Antimicrob Chemother 67:2640–2644. doi:10.1093/jac/dks261. 22782487 PMC3468078

[42] Carattoli A, Zankari E, García-Fernández A, Voldby Larsen M, Lund O, Villa L, Møller Aarestrup F, Hasman H. 2014. In silico detection and typing of plasmids using PlasmidFinder and plasmid multilocus sequence typing. Antimicrob Agents Chemother 58:3895–3903. doi:10.1128/AAC.02412-14. 24777092 PMC4068535

[43] Chen L, Zheng D, Liu B, Yang J, Jin Q. 2016. VFDB 2016: hierarchical and refined dataset for big data analysis–10 years on. Nucleic Acids Res 44:D694–D697. doi:10.1093/nar/gkv1239. 26578559 PMC4702877

[44] Alcock BP, Raphenya AR, Lau TTY, Tsang KK, Bouchard M, Edalatmand A, Huynh W, Nguyen ANV, Cheng AA, Liu S, Min SY, Miroshnichenko A, Tran H, Werfalli RE, Nasir JA, Oloni M, Speicher DJ, Florescu A, Singh B, Faltyn M, Hernandez-Koutoucheva A, Sharma AN, Bordeleau E, Pawlowski AC, Zubyk HL, Dooley D, Griffiths E, Maguire F, Winsor GL, Beiko RG, Brinkman FSL, Hsiao WWL, Domselaar GV, McArthur AG. 2020. CARD 2020: antibiotic resistome surveillance with the comprehensive antibiotic resistance database. Nucleic Acids Res 48:D517–D525. doi:10.1093/nar/gkz935. 31665441 PMC7145624

[45] Didelot X, Wilson DJ. 2015. ClonalFrameML: efficient inference of recombination in whole bacterial genomes. PLoS Comput Biol 11:e1004041-18. doi:10.1371/journal.pcbi.1004041. 25675341 PMC4326465

[46] Stamatakis A. 2014. RAxML version 8: a tool for phylogenetic analysis and post-analysis of large phylogenies. Bioinformatics 30:1312–1313. doi:10.1093/bioinformatics/btu033. 24451623 PMC3998144

[47] Letunic I, Bork P. 2019. Interactive tree of life (iTOL) v4: recent updates and new developments. Nucleic Acids Res 47:W256–W259. doi:10.1093/nar/gkz239. 30931475 PMC6602468

[48] Murray GGR, Wang F, Harrison EM, Paterson GK, Alison E, Harris SR, Holmes MA, Rambaut A, Welch JJ. 2016. The effect of genetic structure on molecular dating and tests for temporal signal. Methods Ecol Evol 7:80–89. doi:10.1111/2041-210X.12466. 27110344 PMC4832290

[49] Didelot X, Croucher NJ, Bentley SD, Harris SR, Wilson J. 2018. Bayesian inference of ancestral dates on bacterial phylogenetic trees. Nucleic Acids Res 46:e134. doi:10.1093/nar/gky783. 30184106 PMC6294524

[50] Wick RR, Judd LM, Gorrie CL, Holt KE. 2017. Unicycler: resolving bacterial genome assemblies from short and long sequencing reads. PLoS Comput Biol 13:e1005595-22. doi:10.1371/journal.pcbi.1005595. 28594827 PMC5481147

[51] Aziz RK, Bartels D, Best AA, DeJongh M, Disz T, Edwards RA, Formsma K, Gerdes S, Glass EM, Kubal M, Meyer F, Olsen GJ, Olson R, Osterman AL, Overbeek RA, McNeil LK, Paarmann D, Paczian T, Parrello B, Pusch GD, Reich C, Stevens R, Vassieva O, Vonstein V, Wilke A, Zagnitko O. 2008. The RAST server: rapid annotations using subsystems technology. BMC Genomics 9:75. doi:10.1186/1471-2164-9-75. 18261238 PMC2265698

[52] Galata V, Fehlmann T, Backes C, Keller A. 2019. PLSDB: a resource of complete bacterial plasmids. Nucleic Acids Res 47:D195–D202. doi:10.1093/nar/gky1050. 30380090 PMC6323999

[53] Alikhan N-F, Petty NK, Ben Zakour NL, Beatson SA. 2011. BLAST ring image generator (BRIG): simple prokaryote genome comparisons. BMC Genomics 12:402. doi:10.1186/1471-2164-12-402. 21824423 PMC3163573

[54] Darling ACE, Mau B, Blattner FR, Perna NT. 2004. Mauve: multiple alignment of conserved genomic sequence with rearrangements. Genome Res 14:1394–1403. doi:10.1101/gr.2289704. 15231754 PMC442156

[55] Langmead B, Salzberg SL. 2012. Fast gapped-read alignment with Bowtie 2. Nat Methods 9:357–359. doi:10.1038/nmeth.1923. 22388286 PMC3322381

