# Supplementary material for: Dissemination of Extended-Spectrum-β-Lactamase-Producing Enterobacter cloacae Complex from a Hospital to the Nearby Environment in Guadeloupe (French West Indies): ST114 Lineage Coding for a Successful IncHI2/ST1 Plasmid
Source: Antimicrob Agents Chemother. 2021 Feb 17;65(3):e02146-20. doi: 10.1128/AAC.02146-20 (PMC8092524; doi:10.1128/AAC.02146-20)
Supplement: Supplemental file 1 [file AAC.02146-20-s0001.pdf]

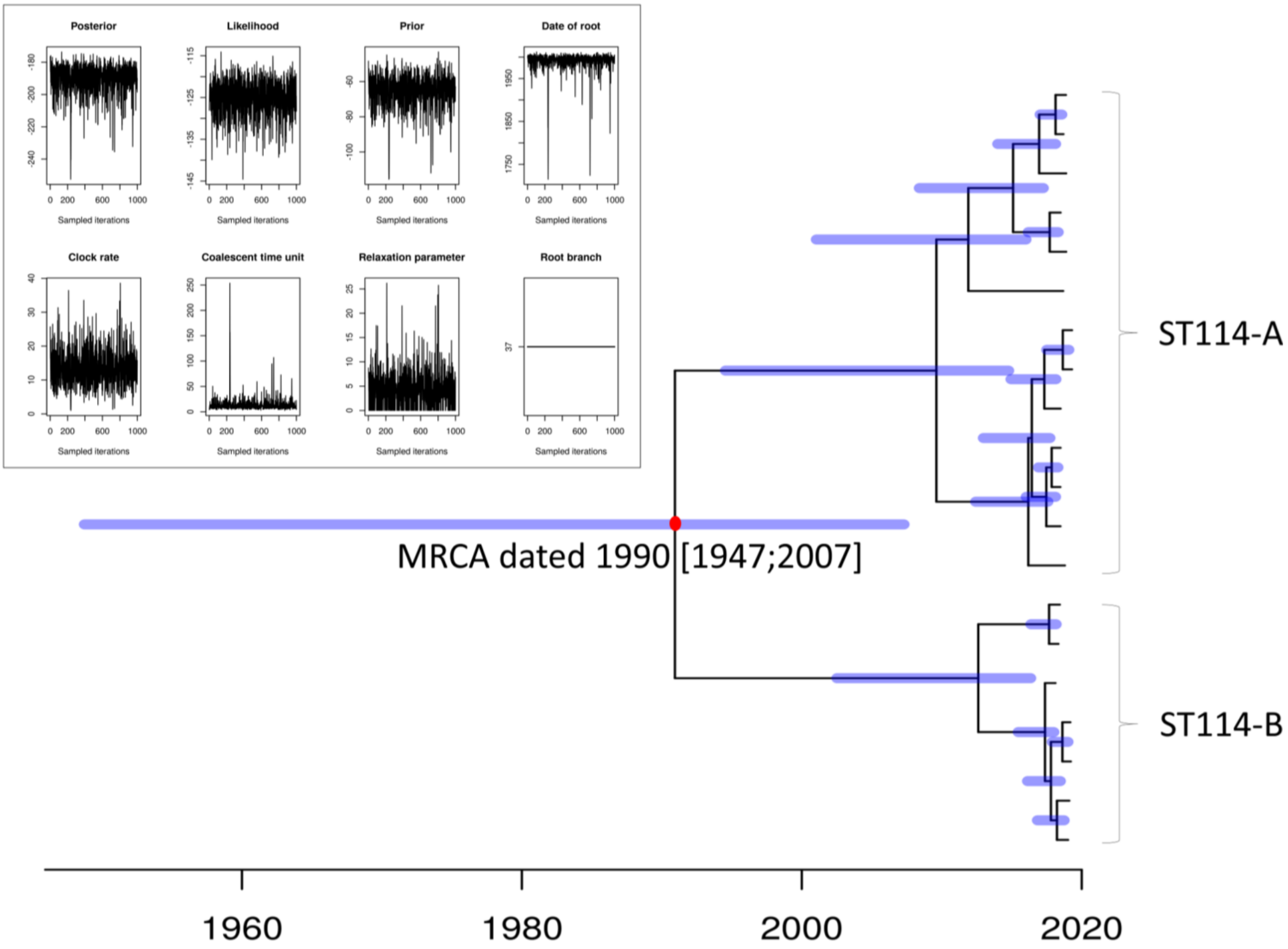

FIG S1. Estimated dated phylogeny of *E. cloacae* complex ST114 in Guadeloupe.

The dated maximum likelihood tree corrected for recombination was designed with BactDating software in a relaxed clock model for 107 iterations. Blue bars indicate 95% credible intervals for nodes dates. The most recent common ancestor (MRCA) was dated to 1990 [1947; 2007]. Convergences of Markov chain Monte Carlo traces for key parameters are indicated in black boxes.

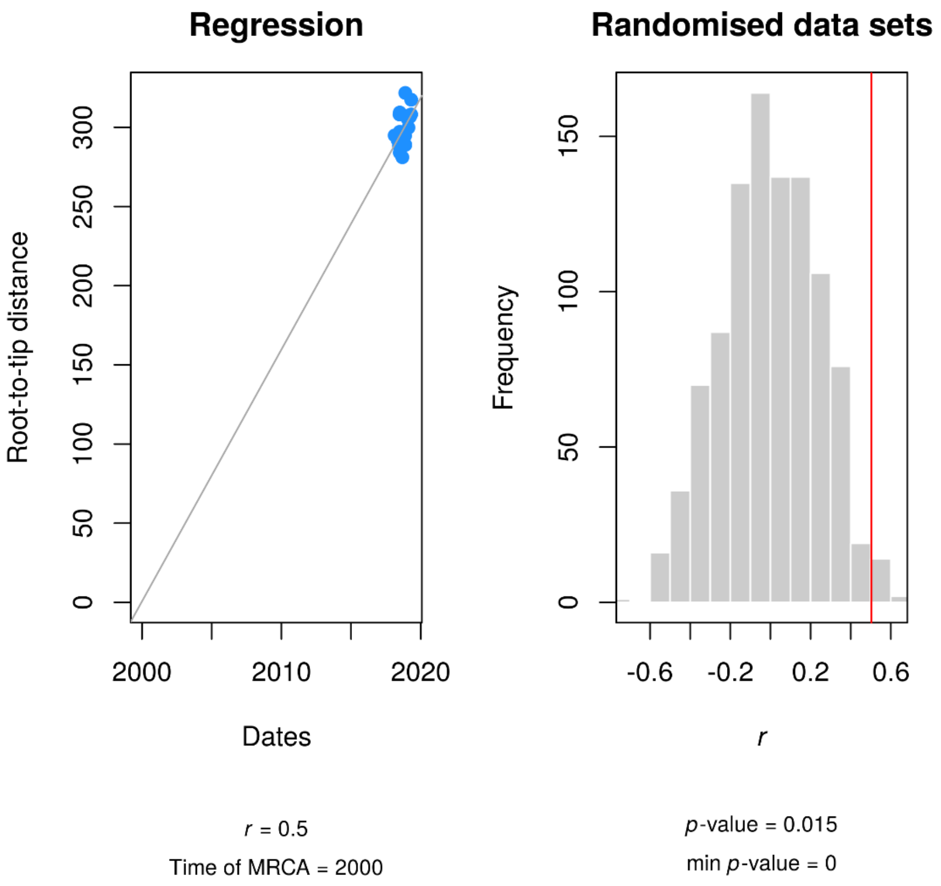

FIG S2. Root-to-tip *vs*. isolation year regression analysis on *E. cloacae* complex ST114. Root-to-tip analysis of real data is presented on the left, giving R2=0.5 and time of most recent common ancestor in 2000 (MRCA). The histogram on the right represents the distribution of R2 values (for the root-to-tip distance vs. year of isolation linear regression) obtained after a total of 1000 random permutations of the isolation year, as suggested by Murray *et al.* (1). The real R2 value is shown as a vertical red line.

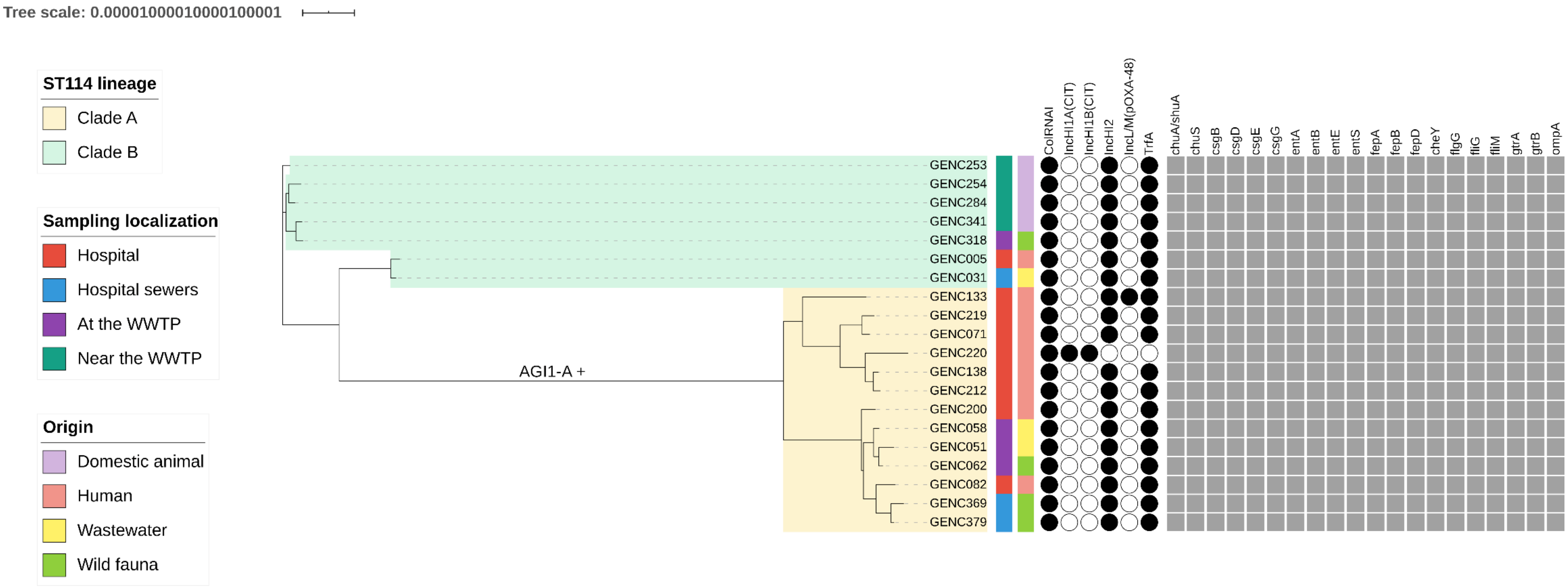

FIG S3. Maximum likelihood phylogenetic tree of *E. cloacae* complex ST114 isolates from Guadeloupe *vs* plasmidic incompatibility groups and virulence genes.

Locations of hosts and sampling sites between the hospital and the wastewater treatment plant (WWTP) are indicated by vertical colored stripes. The tree is subdivided into two lineages, ST114-A (in yellow) and ST114-B (in green). Plasmidic incompatibility groups revealed by PlasmidFinder are represented by black dots; virulence resistance genes characterized by Abricate software and VFDB are indicated by grey squares.

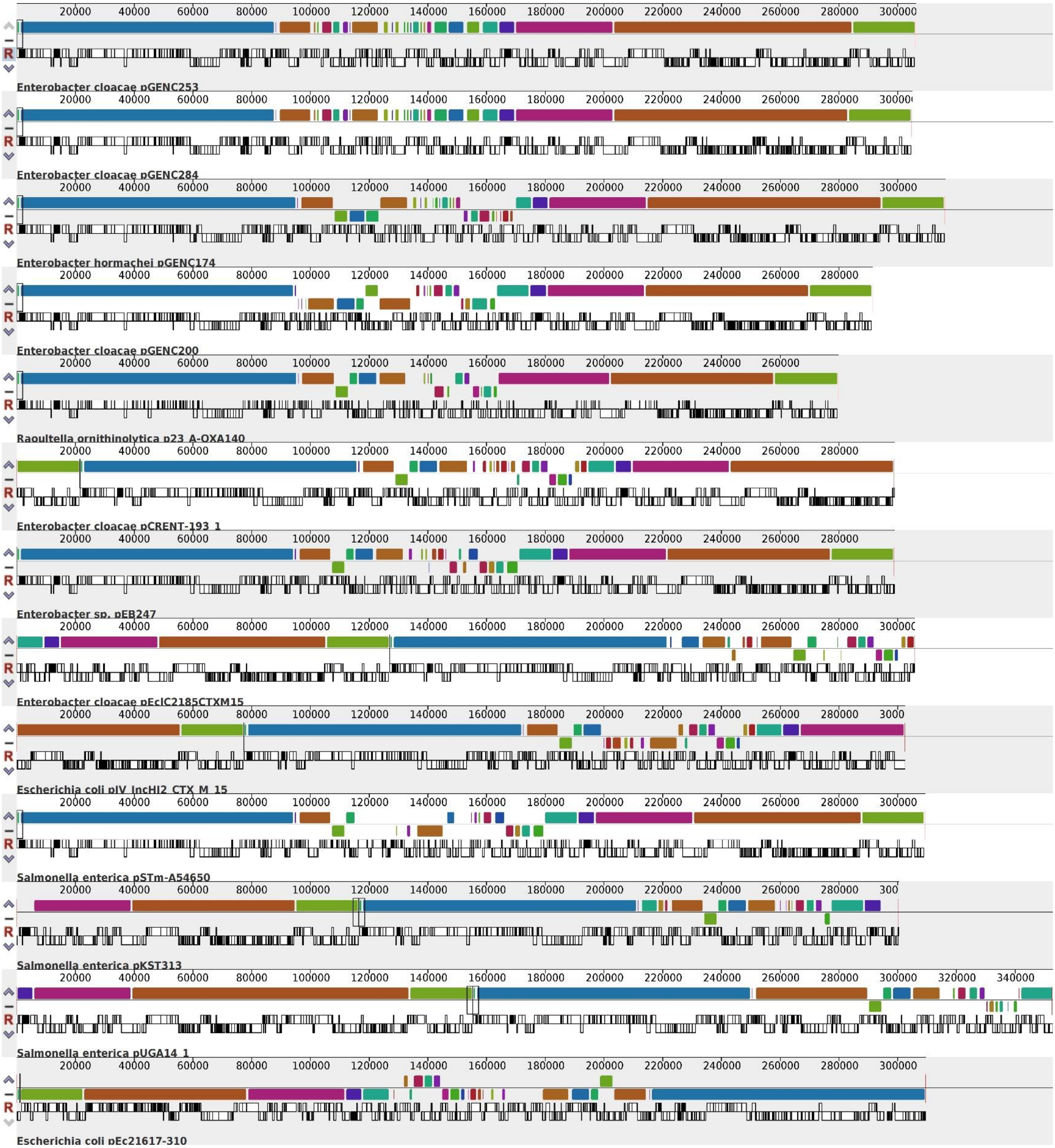

FIG S4. Alignment performed by Mauve software of the four IncHI2/ST1 plasmids from Guadeloupe *vs* nine reference plasmids from the PLSDB database.

This alignment allowed visualization of rearrangements and inversions within plasmids.

TABLE S1. Details of animals and wastewater sampling.

The numbers of strains selected for whole-genome sequencing (WGS) are given for each sampling location between the hospital and the wastewater treatment plant (WWTP) for each host, with the prevalence of extended-spectrum beta-lactamase (ESBL) profiles. Domestic animals are specified by purple squares and wild fauna by green squares.

				***E. cloacae* complex**	
**Sampling type**	**Sampling point – Class**	**Detail**	**Number of sample (N=158)**	**ESBL N (%)**	**WGS selection (N=17)**
**Wastewater**	**Site 1**	Hospital effluents	10	10 (100.0)	2
	**Site 2**	Urban intermediate sampling point	9	6 (66.7)	-
	**Site 3**	WWTP influents	10	7 (70.0)	3
	**Site 4**	WWTP effluents	10	6 (60.0)	2
		**Total**	**39**	**29 (74.4)**	**7**
**Animal**	**Insecta**	Cockroach (*Periplaneta americana*)	29	11 (37.9)	2
	**Reptilia**	Anoles (*Anolis marmoratus speciosus*)	20	1 (5.0)	1
	**Amphibia**	Cane toad (*Rhinella marina*)	19	5 (26.3)	1
	**Aves**	Chicken	11	8 (72.7)	2
		Carib grackle (*Quiscalus lugubris*)	11	1 (9.1)	1
		Goose	2	1 (50.0)	1
	**Mammal**	Pig	10	1 (10.0)	1
		Rat (*Rattus rattus*)	8	1 (12.5)	1
		Dog (*Fila brasileiro*)	6	-	-
		Cow	3	-	-
		**Total**	**119**	**29 (24.4)**	**10**

TABLE S2. Quast comparison of genomic island AGI1-A sequenced from strain GENC200 *vs E. cloacae* complex sublineage ST114-A isolates.

Strain	Mapped lenght (bp)	Unaligned length (bp)	Identity *vs* AGI1-A of GENC200	*aadA1*	*dfrA1*	*sul1*
GENC051	46764	1138	99.930	1	1	1
GENC082	47542	1768	99.905	1	1	1
GENC138	48068	1099	99.887	1	1	1
GENC071	47595	652	99.885	1	1	1
GENC058	48091	1867	99.959	1	1	1
GENC220	44386	0	99.795	1	1	1
GENC062	46753	2320	98.801	1	1	1
GENC219	47476	34	99.468	1	1	1
GENC369	47320	1805	99.412	1	1	1
GENC212	46791	46	99.004	1	1	1
GENC379	47045	1949	98.426	1	1	1
GENC133	47719	1099	99.389	1	1	1

REFERENCE

1. Murray GGR, Wang F, Harrison EM, Paterson GK, Alison E, Harris SR, Holmes MA, Rambaut A, Welch JJ. 2016. The effect of genetic structure on molecular dating and tests for temporal signal. Methods Ecol Evol 7:80–9.
